# Perfectionism, Resilience and Different Ways of Experiencing Sport during COVID-19 Confinement

**DOI:** 10.3390/ijerph19105994

**Published:** 2022-05-15

**Authors:** Juan González-Hernández, Antonino Bianco, Carlos Marques da Silva, Manuel Gómez-López

**Affiliations:** 1 Department of Personality, Evaluation and Psychological Treatment, University of Granada, 18001 Granada, Spain; jgonzalez@ugr.es; 2 Sport and Exercise Sciences Research Unit, Department of Psychology, Educational Science and Human Movement, University of Palermo, 90144 Palermo, Italy; antonino.bianco@unipa.it; 3 Life Quality Research Center (CIEQV), Sport Sciences School of Rio Maior, Polytechnic Institute of Santarém, 2000-044 Santarem, Portugal; csilva@esdrm.ipsantarem.pt; 4 Department of Physical Activity and Sport, Faculty of Sport Sciences, University of Murcia, 30720 Murcia, Spain; 5 Campus of International Excellence “Mare Nostrum”, University of Murcia, 30720 Murcia, Spain

**Keywords:** psychological well-being, adaptive perfectionism, maladaptive perfectionism, resilient resources, athletes, culture, pandemic

## Abstract

The relationship between sports practice and physical and mental health became an important issue during the COVID-19 pandemic, where keeping fit and exercising was one of the best and most popular ways to cope with the confinement situation. The aim of this study was to determine the relationships between perfectionism and resilient resources with psychological well-being, differentiating sports category, gender and experience in a sample of athletes during confinement in different countries affected by the COVID-19 pandemic. An incidental and cross-sectional random sampling method was designed (*n* = 583). The sample was analysed with three different instruments, evaluating perfectionism, resilience and psychological well-being patterns and comparing three groups with different levels of practice due to confinement (full reduction, moderate reduction and only access restrictions). Results show that both male and senior athletes were more organized, resistant to changes and focused their attention and efforts on their demands and potential. They were stimulated by obstacles that required more effort compared to U23, who reported higher concerns and lower organisational scores. Athletes who completely interrupted their sports dynamics showed higher indicators of perfectionism and performed worse in resilience and well-being. Despite this, age and the variability of the athletes’ experiences proved to be relevant factors in an athlete’s trajectory, and continued to represent a certain degree of balance in the face of COVID-19.

## 1. Introduction

Perfectionist research has a long history of study in psychology, both in clinical research and in research on the personality adapted to sport, where the unadaptive or the consequently negative effects (e.g., feelings of failure, indecision, and shame) derived from it have been recognised as a relevant variable for understanding aspects such as depression [1], social phobia [2] or somatic symptoms [3]. On the other hand, it is also interesting how perfectionism in sports situations has shown an ambiguous influence that sometimes has been associated with functional responses (e.g., mental toughness; self-presentation) [4], and in others with dysfunctional ones (e.g., isolation, burnout, exercise dependence) [5,6,7].

The appearance of the COVID-19 disease caused a significant break in everyday life. Given its characteristics, the entire world found itself in an unprecedented situation of confinement and athletes were not exempt from these figures and measures [8]. The state of confinement by COVID-19 proved to be complex to high-performance athletes and it is not yet known how the implemented quarantine measures could affect the physical and psychological aspects of high-performance athletes in the short and long term.

Regular, healthy physical exercise programmes or activities improve mental health, autonomy, memory, speed, body image, and sense of well-being while producing personal stability, improved mood, and improved health. Psychological well-being in sport focuses on the development of skills and personal growth, both conceived as main indicators of positive functioning [9]. In performance-oriented athletes, both perfectionism and resilience work in a similar way, although the circumstances generated by COVID-19 are much more decisive and uncertainties may have diminished many of their hopes and expectations for achieving their sporting goals. It has been hypothesized that in the short term there has not been an acute increase in injuries in professionals, but that in the long term it is unfolding as a burden for high-level sport [10]. However, it will still take time to understand the real impact of COVID-19 pandemic confinement, not only on the physical, but also the mental health of these athletes [11].

Personality is a determining and mediating factor in people who play sports, and a clear difference appears both in those with perfectionist or attention to detail patterns and in those who face adverse situations and difficulties [12]. Usually, people with perfectionist traits tend to worry too much about situations of uncertainty, showing a weak ability to manage stress [13]. On the other hand, people who usually find resources to face adversities (resilient) are more able to tolerate uncertainty and be more positive regardless of the difficulty. The former will live with a lower perception of psychological well-being, while the latter will be more motivated and able to see the future with even greater optimism and hope [14].

Most perfectionist athletes have grown up in environments where love and approval are conditional; so, to feel the love and approval of people they must execute their actions with high levels of perfection. Over high doses of negative self-criticism, any failure or mistake brings with it the risk of being rejected by close contexts of influence (e.g., parents or coaches), thus losing their closeness or affection and feeling that they establish criteria (e.g., expectations) that they cannot meet, with failure meaning a potential loss of contextual acceptance [15,16,17]. Building on high expectations without the proposal of high standards will lead the most perfectionist athletes to be more sensitive to concerns and uncertainties about actions to be taken, leading to emotional (e.g., anxiety), cognitive (e.g., rumination) and behavioural (e.g., control motor) alterations.

Resilience starts to be considered one of the main mental resources in adaptive behaviour towards the processes of change and improvement [18]. It is a variable dependent on several factors such as emotions, supports, experiences, strategies, motivation and self-concept [18,19], based mainly on two concepts, overcoming adversity and positive adaptation [20], with an important component of psychological readjustment [21].

In sports contexts, it is developed from the cognitive, physical and social level to be able to control the threats that may affect the sportsman or woman [22], it influences both personal and sports growth [23]. Their positive relationships with confidence, positive personality, motivation, social support, and concentration act as facilitators for adequate performance in sports performance contexts, or adherence to active lifestyles in healthier orientations. Although, of all these variables the most important for the development of resilience is the belief that the athlete must overcome adversity and the close environment (e.g., family) [24].

Although it is not possible to be too forceful, as resilience is an emergent topic in sports settings [25], resilient resources increase with age [26]. There are studies that show no difference in levels of resilience between men and women [27], and others that point to higher levels of resilience in men, based on a better perception of personal competence [12]. Concerning the sporting experience, the results are also contradictory. Some studies point out that athletes with a higher level of competition are more resilient than amateur athletes [28]. In contrast to this, other literature shows a relationship in which sportsmen and women with less sporting experience have greater resilience [29,30,31]. Morgan et al. [32] presented a study with different focus groups, associating resilience to perceived support among athletes, highlighting their dynamic and systemic qualities that protect from stress, enhancing individual and collective effectiveness. In this sense, the most experienced athletes were indicated to have and to handle greater resilient resources than those indicated by younger or promising athletes.

This study aimed to describe the relationship between perfectionism and resilient resources with psychological well-being. After the literature review, the proposed hypotheses are: (a) worse results of perfectionism and resilience will appear as the time in confinement is longer, (b) more experienced (senior) athletes will show a greater capacity to manage their psychological response during the confinement situation, (c) men will show better indicators in both adaptive perfectionism and resilience and therefore greater psychological well-being, and finally, (d) both women and U_23_ athletes will show greater results in maladaptive perfectionism.

## 2. Materials and Methods

### 2.1. Desing, Participants and Procedure

A transversal, quantitative and non-random study was designed to differentiate the sports category (U23 vs. senior), gender and the practice of sport during the confinement both in South American and Spanish athletes and different levels of impact by the COVID-19 pandemic. Inclusion criteria for participating in the study were: (1) to be part of a Technification Plan of a Sports Federation and to consider competing in sport as their main activity.

A sample of 583 athletes (M = 26.63 years; SD = 6.74) from different cultural backgrounds ((*n*_Spanish_ = 309; 53%); (*n*_South American_ = 274; 47%)) was analysed. The sample distribution by gender included *n*_men_ = 336 (57.6%) and *n*_women_ = 247 (42.4%), and by category including senior athletes (*n* = 300; 51.5%) and U_23_ athletes (*n* = 283; 49.5%). The sample was distributed in: complete reduction in their sports practice during confinement (*n* = 343; 58.8%); moderate reduction (*n* = 154; 26.3%); or only access restrictions in their sports facilities (*n* = 86; 14.9%). Three levels were established when describing the type of confinement suffered by sportsmen and women: (a) full confinement (total confinement, with no possibility of training); (b) moderate confinement, with the possibility of training at home (exceptionally in open or sports facilities, but not in competition), and (c) non-confinement (sportsmen and women who have continued their training in controlled situations, mainly in institutional or private sports facilities). Data collection was carried out during the first half of 2020 in different High-Performance Centres in different countries (Spain, Chile, Costa Rica, Argentina, and Colombia). Before data collection, the following procedure was defined: (a) virtual meetings to request federative permits; (b) sending to athletes (via federation) a letter/document addressed to athletes, explaining the goals and process of the research, including the voluntary nature and the commitment to ethical and confidential compliance. At the same time, measures battery was designed with Google form platform, which was sent to the athletes who agreed to participate, including an informed consent under descriptions of Declaration of Helsinki [33] and the protocol approved by the Ethics Committee (ID: 1494/2020).

### 2.2. Measurement Instruments

Initially, a short questionnaire was designed ad hoc to describe previous considerations of the sample. This included aspects such as gender, age, country, sports experience and how limiting the confinement was in continuing with their sporting lives.

*Perfectionism*. The Spanish adaptation of the Multidimensional Perfectionism Scale (FMPS) [34] by Carrasco et al. [35] is used. It consists of 35 items describing 4 first-order factors (Achievement Expectations, Organization, Fear of Errors and Parental Influences), 2 s-order factors (Adaptive Perfectionism and Maladaptive Perfectionism) and 1 third-order factor (General Perfectionism). The answers cover a Likert scale from “*strongly disagree*” (1) to “*strongly agree*” (5). For the present study, the second-order factors are used, which yielded reliability values for both adaptive perfectionism (α = 0.86) (e.g., “*the organization is very important for me*”) and maladaptive perfectionism (α = 0.83) (e.g., “*The fewer mistakes I make, the more people will want me*“), while the CFA showed a good fit (X^2^/gL = 11.73; *p* = 0.00; CFI = 0.89; NNFI = 0.91; CFI = 0.92; SRMR = 0.05; RMSEA = 0.05).

*Resilience*. The Spanish version of RS-14 Scale [36] was used [37]. This scale is composed of 14 items grouped in two dimensions measuring personal competence [(11) items; self-confidence, independence, decision, resourcefulness and perseverance; (α = 0.78) (e.g., “*I am not afraid to suffer difficulties because I have already experienced them in the past*”), and self and life acceptance (3) items; adaptability, balance, flexibility and a stable life perspective; (α = 0.84) (e.g., “*I am a person with adequate self-esteem*”)]. Responses were collected on a 7-point Likert scale ranging from “*strongly disagreeing*” (1) to “*strongly agreeing*” (7). The internal consistency analysis of the current study for the sample collected has proved satisfactory (α = 0.80), showing an adequate fit (X^2^/gl = 8.51; *p* = 0.01; CFI = 0.91; NNFI = 0.91; CFI = 0.95; SRMR = 0.07; RMSEA = 0.04).

*Psychological well-being*. The Spanish adaptation of Ryff’s psychological well-being scales [38], by Díaz et al. [39] with a Likert orientation of (1) “*strongly disagree*” to (6) “*strongly agree*”, is described by 29 items, grouped into 6 first-order factors (self-acceptance, positive relationships, autonomy, mastery of the environment, personal growth, and purpose with life) and 1 s-order factor (psychological well-being). The second-order factor will be used for this study, which showed a reliability α of 0.89. Confirmatory analysis (CFA) maintains the one-dimensionality of the original version (X^2^/gl = 10.31; *p* = 0.00; CFI = 0.92; NNFI = 0.94; IFC = 0.92; SRMR = 0.06; RMSEA = 0.05). Some examples of items are: “*When I review my life history, I am happy with how things have turned out*”, “*I often feel lonely because I have few close friends with whom to share my concerns*” or “*I tend to worry about what other people think of me*”.

### 2.3. Data Analysis

Descriptive statistical analyses (measures of central tendency, frequencies, homogeneity) and confirmatory factor analyses (CFA) were completed using the following indicators: Chi-square (χ^2^), Akaike Information Criterion (AIC) [40], Comparative Fit Index (CFI), Non-Normed Fit Index (NNFI), Standardized Root-Mean-Square Residual (SRMR) and Root Mean Square Error of Approximation (RMSEA). For an adequate adjustment of the data, the lower the values of X^2^, AIC, NNFI and RMSEA, and the higher those of CFI and NNFI, the more reliable the information obtained would be. The parameter estimation by maximum likelihood (5000 bootstrap samples with bias-corrected confidence intervals 95.00), d Cohen and Cronbach’s alpha are carried out to consider the internal reliability of the instruments and differential analysis. Pearson’s correlation analyses were completed to determine the degree of the linear relationship between the variables under study (controlling for gender and category variables). Finally, the multivariate analysis (MANOVA), for the differential description according to gender and category, was completed. The statistical programme used for these analyses is SPSS (IBM), with version 25.

## 3. Results

Table 1 reflects the descriptive statistics at each of the three levels established when describing the type of confinement suffered by sportsmen and women.

We analysed the calculation of the correlations (“zero-order”) between wellbeing and each one of the other variables, controlling the effect for the gender and category (Table 2). When analysing the linear relationship between the variables studied, it becomes clear that in both the Spanish and South American samples, as age increases (and therefore also the sporting experience), the indicators of resilience and adaptive perfectionism increase significantly (although the links with adaptive perfectionism are poor), while maladaptive perfectionism decreases according to increased sports experience and psychological wellbeing. Concerning the variables between them, adaptive perfectionism shows a positive and significant relationship with personal competence and acceptance, while the relationships are significant and negative with maladaptive perfectionism.

At the same time, in both the Spanish and South American samples, as confinement limitations increased, personal competence (<0.02–0.00) and acceptance (<0.03–0.00) scores worsened, while maladaptive perfectionism (<0.05–0.00) scores increased. Adaptive perfectionism showed no significant relationship in either the Spanish or the South American sample.

To verify the existence of perfectionistic, resilient resources and psychological well-being responses according to gender and category, multivariate contrast analyses were carried out (Table 3). In the sample of Spanish athletes, the results (Pillai’s trace) indicate statistically significant differences in favour of U23 athletes who indicated higher levels of maladaptive perfectionism (F_(5,304)_ = 7.23; *p* = 0.00), while the senior athletes show a moderate magnitude effect (η2 = 0.52; r = 0.58). In the same way, differences appeared in favour of women (F_(5,304)_ = 10.26; *p* < 0.01) against men, with a moderate size effect (η2 = 0.43; r = 0.61). Gender* category interaction was significant (F_(5,304)_ = 9.13; *p* = 0.00) in maladaptive perfectionism, but not in adaptive perfectionism. For resilient resources, both personal competence (F_(5,304)_ = 14.85; *p* = 0.00; η2 = 0.39; r = 0.47) and acceptance (F_(5,304)_ = 16.03; *p* = 0.00; η2 = 0.56; r = 0.61) in male and senior athletes were significantly higher. Psychological well-being showed no significant differences in gender, but did show in category (F_(5,304)_ = 21.24; *p* < 0.01; η2 = 0.48; r = 0.55). Gender*category interaction was significant for personal competence (F_(5,304)_ = 14.21; *p* = 0.00) and acceptance (F_(5,304)_ = 6.91; *p* = 0.00).

In the sample of South American athletes, results (Pillai’s trace) indicate statistically significant differences in favour of U23 athletes who indicated higher levels of maladaptive perfectionism (F_(5,269)_ = 9.42; *p* = 0.00), the opposite of the senior athletes, with moderate magnitude effect (η2 = 0.39; r = 0.51). Similarly, differences appeared in favour of women (F_(5,269)_ = 16.02; *p* = 0.00) against men, with a moderate size effect (η2 = 0.57; r = 0.63). Gender*category interaction was significant (F_(5,269)_ = 15.36; *p* = 0.00) in maladaptive perfectionism, but not in adaptive perfectionism. For resilient resources, both competence perception (F_(5,269)_ = 13.37; *p* = 0.00; η2 = 0.24; r = 0.53) and acceptance (F_(5,269)_ = 14.92; *p* = 0.00; η2 = 0.43; r = 0.64) in male and senior athletes were significantly higher. Psychological well-being showed no significant differences in either gender or category. Gender*category interaction was significant for personal competence (F_(5,269)_ = 17.01; *p* = 0.00) and acceptance (F_(5,269)_ = 13.24; *p* = 0.00).

## 4. Discussion

The present study aimed to describe the relationship and differences between perfectionism, resilient resources, and psychological well-being, according to several sociodemographic characteristics (category, gender, and practice of sport) during the confinement both in South American and Spanish athletes’ cultural contexts being very affected by the COVID-19 pandemic.

Once it was verified that most of the participant athletes went through intense confinement (although those who did not suffer confinement did see their sports routines altered by the restrictions specific to each country (closure of facilities, difficult access to sports materials)), indicators of their psychological response focused on well-being were analysed, establishing linear relationships between perfectionism, resilient resources, and the psychological well-being of the athletes with different elements around their confinement situation. In this sense, it could be verified that those athletes who had more confinement increased their indicators of maladaptive perfectionism, and decreased their resilient resources. However, those with more experience managed their psychological response better by showing fewer indicators of maladaptive perfectionism and better adaptive perfectionism and resilient resources. Thus, and fulfilling the first of the hypotheses raised, as pointed out in the literature that explains the perfectionist functioning [4,6,41,42] and resilient responses in sports contexts [31,43], mainly in the first moments of any destabilizing situation, we should greatly value the sports experience for the development of adaptive resources (functional perfectionism and resilience) in athletes.

In the hope of showing similar results to studies linking them to psychological balance, those athletes who showed higher indicators of adaptive perfectionism (although poorly strong in the relationship) and resilient resources showed significantly higher well-being, while those who showed higher scores of maladaptive perfectionisms indicated lower psychological well-being. Galli & González [24] and other researchers, speak of the importance of resilience for the appearance of positive elements for maintenance in sports practice like commitment [42], self-confidence [43], sociocultural influences [44] coping strategies [16,45], motivation [28] or own well-being [46]. Hill et al. [40] showed in an excellent meta-analytical study that perfectionist concerns show an obvious maladaptive function for athletes, while perfectionist efforts are complex and ambiguous in the first instance, not achieving enough to find a suitable psychological balance. Muñoz-Villena et al. [47] showed in a sample of young people in high-performance sports academies that the main differences between those who showed low and high self-esteem were in the definition of their standards and in the process of orienting themselves to their achievement, while Gaudreau & Verner-Filion [48] found that self-directed perfectionism (setting realistic goals and efforts, and designing a path of coherence) is associated with high levels of positive affection and vitality, as well as greater satisfaction with life than non-perfectionists.

In terms of differences between gender and category, the results agree with the hypotheses. While the more traditional literature on perfectionism in sport describes a broad consensus on the low relevance of gender, the category (more age and sports experience) has been described as important because it shows less perfectionism the higher the sport category [49]. Senior athletes find more positive associations between more adaptive perfectionism and positive affect, and between more maladaptive perfectionism and negative affect, both in favour of the male gender [41].

According to the hypothesis in terms of resilience resources, both personal competition and acceptance are higher for men and top-level athletes. It seems that the confinement situation created by COVID-19 did not change the results obtained in the baseline studies on the influence of gender and category on resilience. Fletcher & Sarkar [21] considered resilience as a construction that is developed and acquired based on personal progress, because of social, psychological, external and internal processes, aspects that are most evident in Olympic athletes who have experienced greater variability in sporting experiences. Bicalho et al. [43] mention that sports resilience is the continuous interaction of individual psychological characteristics and the environment that an athlete may have. On the other hand, Lipowski et al. [50] and González-Hernández et al. [51], in studies with young people, point out that sport practice enhances resilience indicators, as well as being a more protective factor for women than for men. Similarly, it is clear that for both female and U_23_ athletes, higher indicators of maladaptive perfectionism are obtained (greater rigidity of thought, greater concern and self-criticism) similarly with studies conducted with general samples of athletes before the confinement.

Psychological well-being is more significant for senior athletes, with no relevant differences according to gender. While it is understood that more experienced athletes have greater maturity and understanding of life circumstances, younger athletes have been more hopeless about the limitations of confinement. All of this is in line with the hypotheses in studies on psychological well-being among athletes of different ages [16] and sporting experience [52]. Although most of the hypotheses have been confirmed, recent studies have marked the relevance of sports practice for psychological well-being in terms of gender, in favour of men [53], it should be noted that no gender differences have been found between the scores of men and women in this study, although they have shown high indicators of psychological well-being in both subgroups. However, like all the results obtained, the situations created by the COVID-19 confinement should be taken into consideration.

Finally, as expected, given that the situations created by the confinement of COVID-19 have been very similar in both contexts, the results indicate similar results in the Spanish and South American samples.

Although such a study with a large sample size indicates significant differences in athlete samples, it is necessary to point out certain limitations and difficulties in data collection and difficulties in methodological design due to confinement situations. The contact with athletes in this situation has generated a high cost in effort and time of researchers, and data have required a more intense statistical treatment to confirm the validity of the content, and the analysis should be limited to the circumstances generated by COVID-19.

Nevertheless, and for almost the same reasons, due to the similarity of the results obtained, it is evident that the COVID-19 confinement situations had not changed the relations between relevant variables for the psychological response of the athletes. This shows that although many of them have suffered inevitable changes, the study is well planned and carried out, consolidating similar proposals without any alteration for scientific continuity. It would be ideal to contrast these data with the appearance of longitudinal proposals, which would allow us to observe whether such psychological responses would be altered in the long term if the pandemic and the circumstances of confinement persist.

## 5. Conclusions

Athletes are immune to discouragement. Accustomed to suffering, they can look at difficult challenges, facing them and overcoming them by showing superior competences than general population. Undoubtedly, societies are giving them examples to follow in the face of adversity, and in the face of so many limiting circumstances generated by the confinement of the COVID-19 pandemic, they continue to show their usual, functionalised responses to continue their performance and adapt to any change.

While the younger ones will show their usual excess of ambition, the more experienced athletes show greater resources for understanding situations. Most competitive situations had been cancelled (national and international championships, Olympic Games), most of their usual sports dynamics had disappeared or been altered (e.g., training places and facilities, times for travel and sports preparation, contact with colleagues, coaches and rivals), and yet they found new ways to continue with their responsibilities.

Regardless of their gender, it seems that sports maturity made the main difference when it comes to showing both rigidity in perfectionist patterns (both adaptive and maladaptive) and in responding to adversity. Although age, dynamism and variability of the experiences lived by athletes are very relevant factors in the trajectory of an athlete, they continue to represent a degree of balance in front of difficulties of COVID-19.

## Figures and Tables

**Table 1:** Sub-samples distribution differentiating between confinement levels.

*n* = 583	Spanish Athletes (*n* = 309)				South American Athletes (*n* = 274)			
	Gender		Category		Gender		Category	
	Male *n* (%)	Female *n* (%)	U23 *n* (%)	Senior *n* (%)	Male *n* (%)	Female *n* (%)	U23 *n* (%)	Senior *n* (%)
**Full confinement (*n* = 343)**	114 (33.3)	74 (21.6)	92 (26.8)	96 (27.9)	97 (28.8)	58 (16.9)	79 (23)	76 (22.2)
**Moderate confinement (*n* = 154)**	39 (25.3)	34 (22.1)	41 (26.6)	35 (22.7)	42 (27.3)	39 (25.3)	35 (22.7)	43 (27.9)
**Non confinement (*n* = 86)**	23 (26.7)	25 (29.1)	19 (33.7)	26 (66.3)	21 (24.4)	17 (19.8)	17 (19.8)	24 (27.9)

**Table 2:** Correlations between perfectionism, resilience, experience and psychological wellbeing, controlling gender and category (*n* = 583).

	Spanish Athletes				South American Athletes			
	*Perfectionism*		*Resilience*		*Perfectionism*		*Resilience*	
	*PA*	*PM*	*PC*	*A*	*PA*	*PM*	*PC*	*A*
**Limitations of confinement**	0.32	0.53 *	−0.49 *	−0.59 **	0.47	0.58 *	−0.50 *	−0.58 *
**Sport experience**	0.48 **	−0.52 *	0.62 **	0.63 **	0.53 **	−0.39 *	0.65 *	0.54 **
**Psychological Well-being**	0.13 **	−0.42 **	0.44 **	0.69 **	0.18 **	−0.63 **	0.53 **	0.63 **
**Perfectionism Maladaptive**	0.46 **	-	-	-	0.53 **	-	-	-
**Personal Competence**	0.57 **	−0.43 **	-	-	0.45 **	−0.54 **	-	-
**Acceptance**	0.39 *	−0.64 **	0.65 **	-	0.33 *	−0.59 **	0.61 **	-

* si *p* < 0.05; ** si *p* < 0.01; PA = Perfectionism Adaptive; PD = Perfectionism Maladaptive; PC = Personal Competence; A = Acceptance.

**Table 3:** Mean, standard deviations and multivariance analysis, according to gender and category.

	Spanish Sample (*n* = 309)					
	U23		Senior			
	Female (*n* = 28)	Male (*n* = 64)	Female (*n* = 105)	Male (*n* = 112)		
					F_(5,304)_	F_(5,304)_
	*M* (*SD*)	*M* (*SD*)	*M* (*SD*)	*M* (*SD*)	Gender	Category
**Adaptive Perfectionism**	22.65 (1.10)	22.82 (1.73)	24.86 (0.89)	24.91 (0.35)	-	**
**Maladaptive Perfectionism**	25.29 (0.89)	25.28 (0.35)	23.45 (0.14)	23.31 (0.10)	-	**
**Personal Competence**	7.67 (0.14)	8.67 (0.41)	8.92 (0.16)	9.14 (1.20)	*	**
**Acceptance**	8.08 (0.19)	9.11 (0.38)	9.74 (0.15)	10.63 (0.22)	*	*
**Psychological Well-being**	22.87 (0.11)	22.25 (0.22)	24.11 (0.08)	23.94 (0.96)	-	**
	**South American Sample (*n* = 274)**					
	**U23**		**Senior**			
	**Female** **(*n* = 22)**	**Male** **(*n* = 58)**	**Female** **(*n* = 92)**	**Male** **(*n* = 102)**		
					**F_(5,269)_**	**F_(5,269)_**
	***M*** **(*SD*)**	***M*** **(*SD*)**	***M*** **(*SD*)**	***M*** **(*SD*)**	**Gender**	**Category**
**Adaptive Perfectionism**	21.13 (0.85)	21.58 (0.91)	23.62 (0.52)	23.86 (0.68)	-	**
**Maladaptive Perfectionism**	24.04 (0.37)	24.36 (0.72)	23.51 (0.73)	24.47 (0.67)	-	-
**Personal Competence**	8.32 (0.68)	9.95 (0.25)	9.98 (0.67)	10.69 (1.32)	*	**
**Acceptance**	8.16 (0.34)	9.36 (0.52)	10.03 (0.48)	10.75 (0.46)	*	*
**Psychological Well-being**	21.34 (0.38)	23.04 (0.17)	21.87 (0.16)	23.24 (0.53)	-	**

* si *p* < 0.01; ** si *p* < 0.001.

## Data Availability

Data available on request due to restrictions, e.g., privacy or ethical.
